# An examination of the factors associated with male partner attendance in antenatal care in India

**DOI:** 10.1186/s12884-023-05851-8

**Published:** 2023-07-22

**Authors:** Pooja L. Paul, Shanta Pandey

**Affiliations:** 1 Department of Social Work, Umeå University, Umeå, 901 87 Sweden; 2 School of Social Work, Boston College, Chestnut Hill, MA USA

## Abstract

**Background:**

A growing body of literature indicates that including male partners in antenatal care can be instrumental to improving women’s health service utilization and maternal and child health outcomes. Despite this, very few studies have documented overall trends in male partner attendance and what factors influence this involvement within the Indian context. In this study, we used nationally representative data to examine levels of male partner attendance in antenatal care and the factors associated with male partner attendance.

**Methods:**

Data were used from the National Family Health Survey (NFHS-4) conducted in 2015-16. Weighted (probability weights) descriptive statistics were conducted to summarize the level of male partner attendance in antenatal care in India, and multivariable logistic regression models were constructed to estimate the factors associated with male partner attendance in antenatal care.

**Results:**

In 2015, of the women who had attended at least one antenatal care contact during their pregnancy, about 85% reported that their male partners had accompanied them to antenatal care contacts, with variations across regions. Level of education, household wealth, knowledge of pregnancy-related issues, men’s age at marriage, region, and women’s level of autonomy emerged as significant predictors of male partner attendance in antenatal care.

**Conclusions:**

The results of this study highlight the multiple influences that shape male partners’ attendance in antenatal care. The findings underscore the need for a multi-faceted approach to programs and interventions aimed at encouraging male partner involvement; recognizing men both as individuals, as well as being situated within the family/household and community.

## Background

Over the past few decades, there has been increased recognition of the need for male involvement in advancing gender equality and global public health [1, 2]. Moreover, the World Health Organization (WHO) has recommended male involvement during pregnancy, childbirth, and post-birth as a crucial strategy to promote maternal and newborn health [3]. Evidence shows that male involvement^1^ in maternal care is associated with a range of positive outcomes, such as increased odds of antenatal care attendance, facility birth, skill birth attendance, postpartum care, breastfeeding initiation, and decreased odds of maternal depression [1, 2]. Given the importance of male partner involvement for outcomes related to maternal and child health, a body of literature has been devoted to examining what factors predict male partner involvement.

Prior studies show that several factors shape male involvement in maternal care. Globally, men who are older, have higher levels of education, exposure to mass media, better knowledge of pregnancy-related complications [4–8], and those in monogamous marriages [7] were more likely to be involved in maternal care. In contrast, higher levels of women’s autonomy, particularly movement autonomy, were associated with reduced odds of male involvement in maternal care [9]. A study in a rural setting in India documents that social identities, such as caste [10], were also associated with male involvement in maternal care. For instance, the study shows that non-Hindus and those belonging to SC/ST (Schedule Caste/Scheduled Tribe) castes were less likely to accompany their wives for antenatal care than Hindus and men from General castes [10].

Apart from socio-economic factors, broader societal influences such as gender roles and norms also influence male partner involvement. For instance, participants in a qualitative study in Ghana reported that they experienced pressure to embrace the dominant definitions of masculinity and that these limited how men are permitted to engage in pregnancy and childbirth [11]. Similar results were found from a study in Tanzania where men reported that they considered pregnancy-related issues to be a woman’s domain [12]. Qualitative data from India revealed that while some male participants believed it was their responsibility to accompany women to antenatal care visits, others felt they need not be concerned with pregnancy-related issues when they were “busy earning for the family” [13]. Further, health workers’ attitudes was an important determinant of male involvement in maternal care [4]. For instance, a study from the central region of Ghana documented that harsh treatment by healthcare providers discouraged male partners from attending antenatal care clinics [7]. A study from India also found that women often do not want their husbands involved and that the health workers make it difficult for husbands to be involved [4].

In South-Asia, the social construction of gender has a strong influence on maternal health and pregnancy-related outcomes [14–16]. More specifically, in the Indian context, gendered division of reproductive labor often shapes women’s access to maternal care and support during pregnancy. That is, while on the one hand, men are typically not involved in reproductive health as this is seen as a woman’s domain [4, 13]; on the other hand, men are often viewed as the designated gate-keepers and primary decision-makers to women’s health service utilization. For instance, results from the *National Family Health Survey (NFHS-4)* document that 18% of women did not have an institutional delivery because their husbands did not allow them to access services, and 26% of husbands whose wives did go for any antenatal care check-ups felt that it was unnecessary [17].

Thus, within the socio-cultural context of India, male partner attendance during antenatal care contacts can be a strong motivating factor to increase overall awareness and knowledge about maternal care, and increase maternal health service utilization. In this study, we seek to understand male partner attendance in antenatal care (ANC) as an important component of overall male involvement. Although some studies within India have researched male partner attendance in ANC [4, 10, 18], none provide a comprehensive picture of the extent of male partner attendance and the factors associated with male partner attendance. In this study, we used nationally representative data from the National Family Health Survey (NFHS-4, 2015-16) to assess the factors associated with male partner attendance in ANC. We also examine the levels of male partner attendance within the Indian context and the regional variations in the same.

## Methods

### Data source

We analyzed the National Family Health Survey (NFHS-4) data collected in 2015-16 to examine the levels of male partner attendance in ANC and the factors associated with male partner attendance. This is a nationally representative, multi-topic survey undertaken by the International Institute for Population Sciences and Macro International [17]. The sampling design was a stratified two-stage sample, and the 2011 census was used as the sampling frame. For each state, urban and rural samples were drawn separately and proportionate to the state. Data were available for twenty-nine states and seven union territories (an additional state of Telangana was added in the fourth round of the NFHS survey, NFHS-4 2015-16). Overall, 723,875 eligible women (15–49 years old) and 122,051 eligible men (15–54 years old) were identified for the survey. A sample of 699,686 women and 112,122 men completed interviews. We linked data from men’s questionnaire with data from women’s questionnaire to create a couples dataset using identification numbers for the primary sampling unit (PSU), household, and line number. We also linked data on household wealth index, region, place of residence, and distance to health facility from the household questionnaire with the couples dataset. From the couples data, we excluded nulliparous women and those who had given birth five years before the survey. Further, women who reported having zero antenatal care contacts (NFHS-4: 3,729), those with missing data on antenatal care contacts (NFHS-4: 208), and women who had missing data on male partner attendance in ANC were excluded from the analysis. Thus, to examine the levels of male partner attendance in ANC the analytic sample included data on 20,177 couples. Additionally, when examining the determinants of male partner attendance in ANC, respondents were dropped if they had missing data on the socio-demographic control variables (caste, occupation, age at marriage, and pregnancy complications). The final analytic sample included data on 18,868 couples.

### Research ethics

In alignment with the ethical guidelines, permission was obtained from the Boston College Institutional Review Board (IRB) for the study.

### Measures

#### Dependent variable

***Male partner attendance in ANC***: We recoded male partner attendance in ANC based on the survey question: *“Was the child’s father present at any antenatal care contact for your most recent child?”*; women who answered yes were coded as 1; otherwise as 0.

#### Independent variables

To examine the factors predicting male partner attendance in ANC, we used data from the NFHS-4 (NFHS-4, 2015-16). Education, caste^2^, religion, age, age at marriage, work status, knowledge of pregnancy-related complications, pregnancy complications, number of children ever born, and women’s autonomy were measured at the individual level. Household wealth, family type, place of residence, region, and distance to health facility were measured at the household level. Detailed information on coding and management of variables provided in Table 1.

**Table 1:** Definition of variables used in the study

NFHS Survey questions	NFHS response categories	Recoded response categories
Men’s Characteristics		
*Educational level*	Variable included five categories: no education, primary education (pre-primary to the completion of 5th grade of schooling), secondary education (6th grade to the completion of 10th grade); higher secondary and above higher secondary (beyond 10th grade). No education will be used as the reference category.	1 = no education; 2 = primary education; 3 = secondary education; 4 = higher secondary and above higher secondary (beyond 10th grade).
*Caste*	Variable included five categories: scheduled caste, scheduled tribe, other backward classes, none of them, don’t know. ‘None of them’ will be used as the reference category.	1 = scheduled caste; 2 = scheduled tribe; 3 = other backward classes; 4 = none of them
*Religion*	Variable included nine categories: Hindu, Muslim, Christian, Sikh, Buddhist, Jain, Jewish, No religion, Other. Hindu will be used as the reference category.	1 = Hindu; 2 = Muslim; 3 = Others
*Age*	Continuous variable representing respondent’s age.	0 = 15–24 years’ old; 1 = 25 and above
*Age at marriage*	Continuous variable representing respondent’s age at marriage.	0 = Men married below 18 years of age; 1 = Men married at 18 years of age or older coded as 1.
*Work status*	Based on the question regarding whether the respondent is currently working.	0 = No work; 1 = Currently employed
*Knowledge of pregnancy-related issues*		Score created based on whether a health worker spoke to the male respondent on the following topics during the pregnancy: bleeding, convulsions, prolonged labour. The index includes 3 questions, and the score will range from 0 to 3
**Women’s Characteristics**		
*Male attendance at antenatal care* “Was your husband present during any antenatal check-up for your most recent child?”	Responses categories: Yes; No	0 = No; 1 = Yes
*Educational level*	Variable included five categories: no education, primary education (pre-primary to the completion of 5th grade of schooling), secondary education (6th grade to the completion of 10th grade); Higher secondary and above higher secondary (beyond 10th grade). No education will be used as the reference category.	1 = no education; 2 = primary education; 3 = secondary education; 4 = Higher secondary and above higher secondary (beyond 10th grade).
*Age*	Continuous variable representing respondent’s age.	Used without recoding
*# of children ever born*	Continuous variable representing number of children	0 = None; 1 = At least one child
*Pregnancy complications*	Variable created based on response (yes/no) to any one of the following survey questions: During pregnancy, had difficulty with daylight vision; during pregnancy had swelling on legs, body, face; had convulsions not from fever. Respondents who answered yes to any of these questions were coded as 1, otherwise as 0.	0 = No complications 1 = Had at least one complication during pregnancy
*Women’s Autonomy*	Adapted from Thapa et al. (2013)* ● People who usually decides on respondent’s healthcare ● People who usually decides on large household purchases ● People who usually decides on visits to family and relatives. ● Whether woman is allowed to go to health facility; marketplace and outside the community ● Has a bank/savings account. Has money that the respondent alone can decide to use.	Score will be created from responses to each of the questions, and the score will range from 0 to 4.
**Household characteristics**		
*Wealth quintile* ^*a*^	This variable includes five categories: Poorest, Poorer, Middle, Richer, Richest.	1 = Poorest; 2 = Poorer; 3 = Middle; 4 = Richer; 5 = Richest
*Family type*	Based on the question, what is your relationship to the household head.	0 = Joint family (those who reported any other relationship to household head); 1 = Nuclear family (those who reported themselves or wife as household head)
*Place of residence*	This variable included two categories: Rural, Urban	0 = Urban; 1 = Rural
*Region*	State variable will be recoded into regions based on categorization of National Family and Health Survey-4 (IIPS, 2017). South will be used as the reference category.	1 = North (Chandigarh, Delhi, Haryana, Himachal Pradesh, Jammu & Kashmir, Punjab, Rajasthan, Uttarakhand) 2 = Central (Chattisgarh, Madhya Pradesh, Uttar Pradesh) 3 = East (Bihar, Jharkhand, Odisha, West Bengal) 4 = North East (Arunachal Pradesh, Assam, Manipur, Meghalaya, Mizoram, Nagaland, Sikkim, Tripura) 5 = West (Dadar & Nagar Haveli, Daman & Diu, Goa, Gujarat, Maharashtra) 6 = South (Andaman & Nicobar Islands, Andhra Pradesh, Karnataka, Kerala, Lakshadweep, Puducherry, Tamil Nadu, Telangana)
*Distance to health facility*	This variable included two categories: Distance to health facility is not a problem, is a big problem	0 = Distance to Health facility not a problem 1 = Distance to Health facility is a big problem

### Statistical analyses

We conducted weighted (probability weights) descriptive statistics to summarize the level of male partner attendance in ANC in India, overall, and by state and region.

To examine the factors influencing male partner attendance in ANC, we employed logistic regression analyses. Data on all twenty-nine states and seven union territories were included (The state of Telangana was formed on June 2, 2014 and was previously a part of Andhra Pradesh. With Telangana, there are now 29 states and eight union territories in India). Multivariable logistic regression models were employed to estimate the relationship between the predictor variables and male partner attendance, and Odds Ratio (OR) and 95% confidence interval (CI) have been reported. The associations were deemed significant at p < 0.05.

We applied Stata SE version 14.2 to analyze the data. To account for the complex sample design and to obtain cluster-robust standard errors, we used survey weights to obtain representative estimates.

## Results

Table 2 presents the prevalence of male partner attendance in ANC. Overall in 2015, of women who reported that they had attended at least one antenatal care contact, about 85% reported that their male partners had accompanied them to antenatal care contacts. In 2015-16, states with high levels of male partner attendance were Tripura (95%), Kerala (94%), Sikkim (93%), Tamil Nadu (92%), and West Bengal (91%). Overall, in 2015, the Southern (88%) and Eastern (87%) regions showed the highest levels of male partner attendance. States with the lowest levels of male partner attendance were Mizoram (58%), Meghalaya (62%), Nagaland (66%), Arunachal Pradesh (75%), and Uttar Pradesh (76%).

**Table 2:** Trends in male partner attendance in antenatal care, NFHS-4, 2015-16 (N = 20,177)

State	Male partner present (%)	State	Male partner present (%)
India	85.35		
Andhra Pradesh	85.92	Nagaland	66.18
Assam	82.13	Orissa	90.37
Bihar	81.24	Punjab	89.79
Goa	90.63	Rajasthan	80.75
Gujarat	85.38	Tamil Nadu	92.32
Haryana	89.43	Telangana	86.36
Himachal Pradesh	89.05	West Bengal	91.98
Jammu	90.72	Uttar Pradesh	76.83
Karnataka	83.46	New Delhi	87.19
Kerala	94.81	Arunachal Pradesh	75.68
Madhya Pradesh	81.33	Tripura	95.63
Maharashtra	85.32	Uttaranchal	86.21
Manipur	79.79	Sikkim	93.39
Meghalaya	62.24	Jharkhand	81.48
Mizoram	58.80	Chhattisgarh	90.05
**Regions**			
North	86.15		
Central	79.82		
East	87.40		
North-East	81.49		
West	85.48		
South	88.91		

The socio-demographic characteristics of the respondents are presented in Table 3. About 85% of women reported that the male partner was present during antenatal care contacts for their last child. Among women respondents, 51% reported having completed secondary education (up to 10th grade) and 13% reported completing primary education, with 21% of women reporting that they received no formal education. The average age of women respondents was 26 years. The majority of men (56%) had completed secondary level education (up to 10th grade), and 16% had completed higher education. Mens’ age varied from 17 to 54 years, with 91% of the men in the age group of 25 years and above. Most men also reported that they were married at 18 or more years (93.3%). A large proportion of the men were Hindu (82%), and 44% belonged to Other Backward Classes (OBC). The couples had two children on average; for approximately 34% of couples, this was their first child ever-born. Further, more than half of the households were rural (64%) and belonged to the Central (22%) and Southern (22%) regions.

**Table 3:** Respondents’ Individual and Household level characteristics (N = 18,868)

	Full sample (%)
*Individual level variables *	
*Men’s Characteristics*	
**Educational level**	
No formal Education	12.67
Primary	14.19
Secondary	56.67
Higher	16.48
**Religion**	
Hindu	82.17
Muslim	12.68
Others	5.15
**Caste**	
Scheduled Castes (SC)	20.81
Scheduled Tribe (ST)	11.01
Other Backward Classes (OBC)	44.84
General	23.34
**Age**	
15–24 years	8.61
25 years and above	91.39
**Age at marriage**	
Less than 18 years	6.69
18 years and older	93.31
**Work status**	
Unemployed	6.62
Currently employed	93.38
**Male respondent’s Mean Knowledge of pregnancy-related issues (Mean, SD)**	1.58 (1.35)
*Women’s Characteristics*	
**Educational level**	
No education	21.69
Primary	12.73
Secondary	51.86
Higher	13.72
**Age (Mean, SD)**	26.87 (0.05)
**Number of children ever born**	
None	34.25
At least one child	65.75
**Complications during pregnancy**	
No	58.07
Yes	41.93
**Level of Autonomy (Mean, SD)**	2.55 (1.06)
*Household level variables*	
**Household Wealth Index**	
Poorest	16.86
Poorer	19.25
Middle	21.65
Richer	21.17
Richest	21.07
**Family Type**	
Joint	43.34
Nuclear	56.66
**Place of residence**	
Urban	35.37
Rural	64.63
**Region**	
North	13.84
Central	22.66
East	19.65
North-East	3.45
West	17.59
South	22.80
**Distance to health facility**	
Not a problem	78.18
Big problem	27.82

Table 4 presents the results from the multivariable regression models examining the factors influencing male partner attendance in ANC. In Model I, results revealed that higher education and household wealth, increased knowledge of pregnancy-related issues, being married at 18 years and older, and higher level of women’s autonomy were significantly associated with increased odds of male partner attendance in antenatal care. Women with higher education (OR = 1.66, 95% CI [1.22–2.25]) and secondary education (OR = 1.38, 95% CI [1.18–1.62]) were more likely to report that their male partner accompanied them to antenatal care contacts, as compared to women with no education. While men with higher education were more likely to attend antenatal care contacts than men without formal education, this result was not statistically significant. Male partner’s knowledge of pregnancy-related issues was also associated with attendance at antenatal care; that is, every unit increase in knowledge of pregnancy-related issues was associated with a 13% increased likelihood of attending antenatal care contacts (OR = 1.13, 95% CI [1.08–1.18]). Further, men belonging to the richest wealth quintile households were more likely to attend antenatal care contacts than those belonging to the poorest wealth quintile (OR = 1.58, 95% CI [1.19–2.11]). While respondent’s age was not associated with male partner attendance, age at marriage was significantly associated such that men married at 18 years and older were 31% more likely to be present at antenatal care (OR = 1.31, 95% CI [1.00–1.56]) compared with those married before attaining 18 years of age. Further, compared to respondents belonging to the Southern region, those from the North (OR = 0.74, 95% CI [0.60–0.92]), Central (OR = 0.61, 95% CI [0.51–0.73]), North-East (OR = 0.59, 95% CI [0.46–0.76]) and Western region (OR = 0.67, 95% CI [0.53–0.86]) were significantly less likely to report male partner attendance at antenatal care contacts.

**Table 4:** Multivariable logistic regressions predicting male partner attendance in antenatal care: Adjusted Odds Ratios (AOR) at 95% Confidence Interval (CI)

	Model I		Model II	
	Male partner attendance N = 18,868		Male partner attendance (adjusted for women’s autonomy) N = 18,868	
	AOR	CI	AOR	CI
*Individual level variables*				
*Men’s characteristics*				
**Education (ref: No formal education)**				
Primary	1.072	(0.877–1.309)	1.069	(0.875–1.307)
Secondary	1.143	(0.957–1.365)	1.149	(0.962–1.371)
Higher	1.283	(0.974–1.690)	1.293	(0.983–1.702)
**Religion (ref: Hindu)**				
Muslim	1.026	(0.845–1.246)	1.050	(0.862–1.278)
Others	0.928	(0.670–1.287)	0.922	(0.663–1.282)
**Caste (ref: General)**				
Scheduled Castes (SC)	1.147	(0.954–1.378)	1.138	(0.946–1.368)
Scheduled Tribe (ST)	0.912	(0.784–1.060)	0.920	(0.792–1.070)
Other Backward Classes (OBC)	1.051	(0.860–1.286)	1.066	(0.872–1.303)
**Age (ref: 25 years and above)**				
15–24 years	0.975	(0.789–1.206)	0.959	(0.775–1.187)
**Age at marriage (Less than 18 years)**				
18 years and older	1.312**	(1.100–1.566)	1.304**	(1.093–1.555)
**Work status (ref: Unemployed)**				
Currently employed	0.923	(0.734–1.162)	0.904	(0.717–1.141)
**Knowledge of pregnancy-related issues**	1.132***	(1.085–1.182)	1.131***	(1.084–1.181)
*Women’s characteristics*				
**Education (ref: No formal education)**				
Primary	1.016	(0.854–1.209)	0.997	(0.837–1.187)
Secondary	1.383**	(1.183–1.617)	1.335***	(1.141–1.561)
Higher	1.661**	(1.225–2.251)	1.529**	(1.131–2.068)
**Age**	1.005	(0.992–1.018)	1.002	(0.989–1.015)
**Number of children ever-born (ref: None)**				
At least one child	0.981	(0.844–1.141)	0.983	(0.846–1.143)
**Complications during pregnancy (ref: No)**				
Yes	1.065	(0.951–1.193)	1.065	(0.951–1.193)
**Level of autonomy**			1.147**	(1.075–1.223)
*Household level variables*				
**Household Wealth Index (ref: Poorest)**				
Poorer	1.103	(0.940–1.293)	1.100	(0.937–1.290)
Middle	1.228*	(1.026–1.471)	1.218*	(1.016–1.459)
Richer	1.361**	(1.093–1.695)	1.350**	(1.083–1.683)
Richest	1.585**	(1.190–2.110)	1.558**	(1.170–2.073)
**Family Type (ref: Joint)**				
Nuclear	1.079	(0.949–1.228)	1.043	(0.917–1.187)
**Place of residence (ref: Urban)**				
Rural	0.962	(0.821–1.127)	0.972	(0.828–1.140)
**Region (ref: South)**				
North	0.744**	(0.598–0.925)	0.746**	(0.600–0.929)
Central	0.609***	(0.506–0.734)	0.621***	(0.515–0.750)
East	1.033	(0.833–1.281)	1.051	(0.846–1.306)
North-East	0.592***	(0.464–0.757)	0.588***	(0.460–0.752)
West	0.675**	(0.528–0.863)	0.687**	(0.538–0.879)
**Distance to health facility (ref: Not a problem)**				
Big problem	0.946	(0.822–1.089)	0.959	(0.835–1.101)
Constant	2.677***	(1.594–4.495)	2.209***	(1.300–3.756)

***p < 0.001, ** p < 0.01, * p < 0.05

Our results show that religion, caste, family type, age, work status, pregnancy complications, number of children ever-born, place of residence, and distance to health facility had no association with odds of male partner attendance in ANC when controlling for all other factors.

In Model II, we examined the factors predicting male partner attendance in antenatal care while controlling for women’s autonomy. We found that women’s autonomy was significantly associated with male partner attendance (see Table 4). With every unit increase in score for women’s autonomy, there was a 15% increased likelihood that the male partner was present during antenatal care contacts (OR = 1.15, 95% CI [1.08–1.22]).

## Discussion

This study uses nationally representative data to understand the levels of male partner attendance in ANC, and the factors associated with male partner attendance. The findings show that overall about 85% of men were present for at least one antenatal care contact during the pregnancy for their last child, with variation by state. The results from the regression analyses indicate that education, household wealth, region, knowledge of pregnancy-related issues, age at marriage, and women’s level of autonomy were significant predictors of male partner attendance in ANC.

The results from the multivariable analysis revealed that men with better knowledge of pregnancy-related issues were more likely to be involved in maternal care. This is consistent with previous research in South Asia [13, 18]. Men with better knowledge of maternal care and pregnancy complications may also be in a better position to be involved in decision making regarding antenatal care and place of delivery and to advocate for a health facility birth, increasing maternal health service utilization among women [18]. It is important to note that knowledge of pregnancy complications and pregnancy preparedness shares a reciprocal relationship with attending antenatal care contacts. While better knowledge leads to greater odds of male partner attendance, research shows that men accompanying their partners to antenatal care also reported higher birth preparedness and readiness regarding pregnancy complications [19]. Within this context, strategies to ensure that men accompany women on at least one antenatal check-up can provide an important opportunity for them to be counseled on maternal health issues. Educating men on pregnancy issues can also be crucial to fighting cultural taboos around pregnancy, increasing joint decision-making on health issues, and improving intra-spousal communication [20]. Interestingly, women’s education was positively associated with male partner attendance in antenatal care, indicating that educated women could encourage men’s greater involvement in joint decision-making about maternal care.

We also note that overall household wealth is positively associated with male partner attendance in ANC. While on one hand, higher levels of household wealth are associated with increased male partner attendance; on the other hand, it is important to highlight here that this may be a crucial barrier to attendance for individuals belonging to households within the lower wealth quintiles or those that work in the informal sector. For instance, for individuals that are employed as informal sector workers or daily wage earners, accompanying their partner to ANC may result in considerable financial loss.

An unanticipated finding was a significant and positive relationship between women’s autonomy and male partner attendance. Within the Indian context, women’s autonomy can strongly influence healthcare decision-making. According to the latest NFHS-4 report, only about 42% of women in India were financially independent, about 53% of women had a bank account that they alone could use, and further, only 41% were allowed to go to the market, health facility, or outside of their village/ community by themselves [17]. Despite the strong influence that women’s autonomy may have on their health-seeking behavior and on male participation, few studies have included women’s autonomy as a predictor of male partner attendance in antenatal care. Prior research that explores this association shows that women’s autonomy has an inverse relationship with male partner attendance in antenatal care [9]. This evidence suggests that male partners of women with more freedom of movement and financial autonomy would be less likely to accompany them to antenatal care contacts; thus, in such cases, the male partner’s involvement would be an expression of women’s lack of autonomy. In contrast, the results of this study show that every unit increase in women’s level of autonomy was associated with an increased likelihood that their male partners would accompany them to antenatal care contacts. According to the previously mentioned study [9], while increased financial and movement autonomy would improve healthcare accessibility for women, this may not necessarily suggest that this would lead to greater involvement of men in maternal care. However, the significant positive association found between women’s autonomy and male partner attendance in this study underscores the potential of women’s empowerment as a critical strategy to improve male partner attendance as well as maternal health service utilization. Further research should be undertaken to examine whether women’s autonomy can have a reinforcing influence on various aspects of male involvement in maternal care.

Finally, this study found that controlling for all other factors, age, family type, work status, and number of children ever-born had no association with male partner attendance in antenatal care. This outcome is contrary to that of earlier studies which show that older men [5], those with fewer children [7], and those that lived in nuclear families [10, 13] were more likely to accompany their wives to antenatal care visits. Furthermore, our results also show that caste, religion, and place of residence (rural/urban) is not associated with male partner attendance, when all other factors are controlled for. However, it is interesting to note that region emerges as an important predictor. Male partner attendance was significantly lower in Northern, Central, North-eastern, and Western regions than in the Southern region. Given that the Central, Eastern, and North-Eastern regions show some of the worst indicators in maternal health service utilization [21], encouraging male partner involvement in these regions could be a potential strategy to improve maternal health outcomes. Further research is needed to acquire a better understanding of the cultural and social contexts in these regions that may present barriers to male partner attendance in ANC.

### Limitations

This study has some limitations. Due to limited questions regarding male partner attendance in the survey, the study cannot account for male partner attendance at multiple antenatal care contacts or sustained male involvement. Thus, it was not possible to make a distinction between low and high levels of male partner attendance in antenatal care. It may also be useful to explore other aspects of male involvement, including providing financial support, arranging transportation, and presence at delivery and postpartum care. Due to data limitations, we could not account for the influence of prevalent gender roles and norms, as well as health system-level factors such as harsh treatment from health providers, which might impede male partner attendance in antenatal care. Additionally, it is important to consider the potential for social desirability bias which could lead to an overestimation of reports of male partner attendance.

## Conclusions

Despite these limitations, the findings contribute to literature by documenting the levels of male partner attendance in ANC in India, and the multiple influences that shape male partners’ attendance. These include factors such as education, household wealth, region, knowledge of pregnancy-related issues, age at marriage, and women’s level of autonomy. The findings from this study also highlight that the levels of male partner attendance during antenatal care varies across regions suggesting the need for interventions that are context-specific and community-based.

Overall, these findings suggest that strategies based on knowledge building, such as providing information resources, mass-media campaigns, and workshops on counseling both men and couples within communities can be useful in encouraging male partner involvement in antenatal care. Social workers and community health workers can be instrumental in implementing such community-outreach interventions that target not only male partners of women but also other male members within the community, such as community leaders and religious or village heads. More specifically, male community health workers/ Male Health Activists can help engage with men on maternal and child health issues [22]. That being said, while male partner attendance in ANC should be encouraged, it is crucial to note that this must not be viewed as a prerequisite for women receiving care.

Apart from examining the factors associated with male partner attendance in antenatal care, this is one of the first studies that presents the levels of male partner attendance in India at a national and regional level, thus serving as a primer for future research. Further qualitative research can also provide a more in-depth understanding of the regional variations in male partner attendance. It would be useful for future studies to provide insights into sustained male partner attendance throughout the antenatal period, as well as to understand male partners’ perception of attendance in antenatal care, particularly their experiences of negotiating prevalent gender norms and health workers’ attitudes.

## Data Availability

The datasets generated and/or analysed during the current study are available in the Demographic and Health Survey repository. Retrieved from: https://dhsprogram.com/data/dataset/India_Standard-DHS_2015.cfm?flag=0.

## Footnotes

1 Within this context, male involvement in maternal care is used as an umbrella term and includes male partner attendance in antenatal care, arrangement of transportation, assistance in household activities, presence during delivery and postpartum care.

2 Marginalized Hindu Communities were classified as Scheduled Castes (SC), Scheduled Tribes (ST) and Other Backward Classes (OBC) based on the Constitution (Scheduled Castes) Order, 1950.
